# Supplementary material for: Size-dependent activity and selectivity of carbon dioxide photocatalytic reduction over platinum nanoparticles
Source: Nat Commun. 2018 Mar 28;9:1252. doi: 10.1038/s41467-018-03666-2 (PMC5871894; doi:10.1038/s41467-018-03666-2)
Supplement: Supplementary file 1 — Supplementary Information(PDF 1788 kb) [file 41467_2018_3666_MOESM1_ESM.pdf]

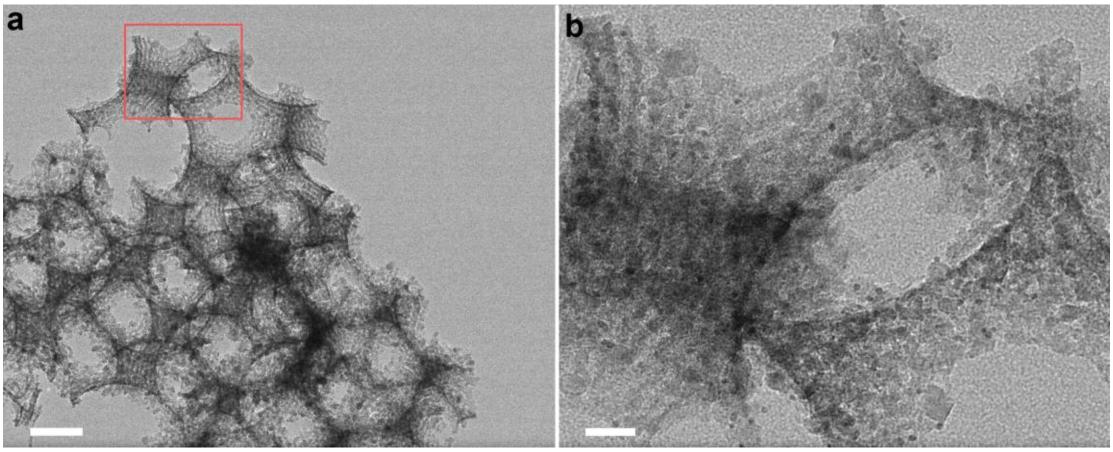

Supplementary Figure 1 | TEM analysis. a TEM and **b** HR-TEM images of the Pt/HTSO, the NaCl-EG solution was used instead of HCl-EG solution in the 7.0PHTSO synthesis process, the red square denotes as the magnification of the local position. The scale bar is 100nm in **a** and 20nm in **b**.

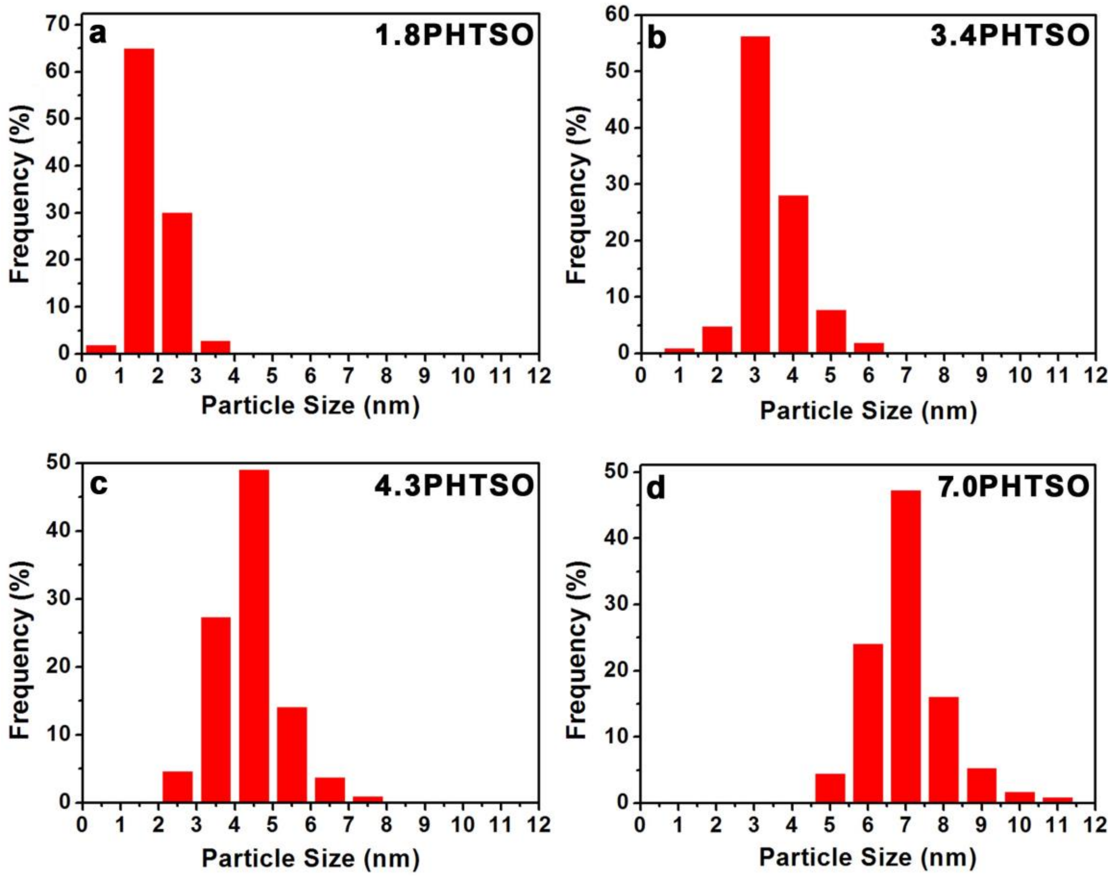

Supplementary Figure 2 | Size distribution statistics of variable sized Pt NPs. a-d

The histogram of size distribution statistics of Pt NPs in *x*PHTSO (*x* = 1.8, 3.4, 4.3 and 7.0).

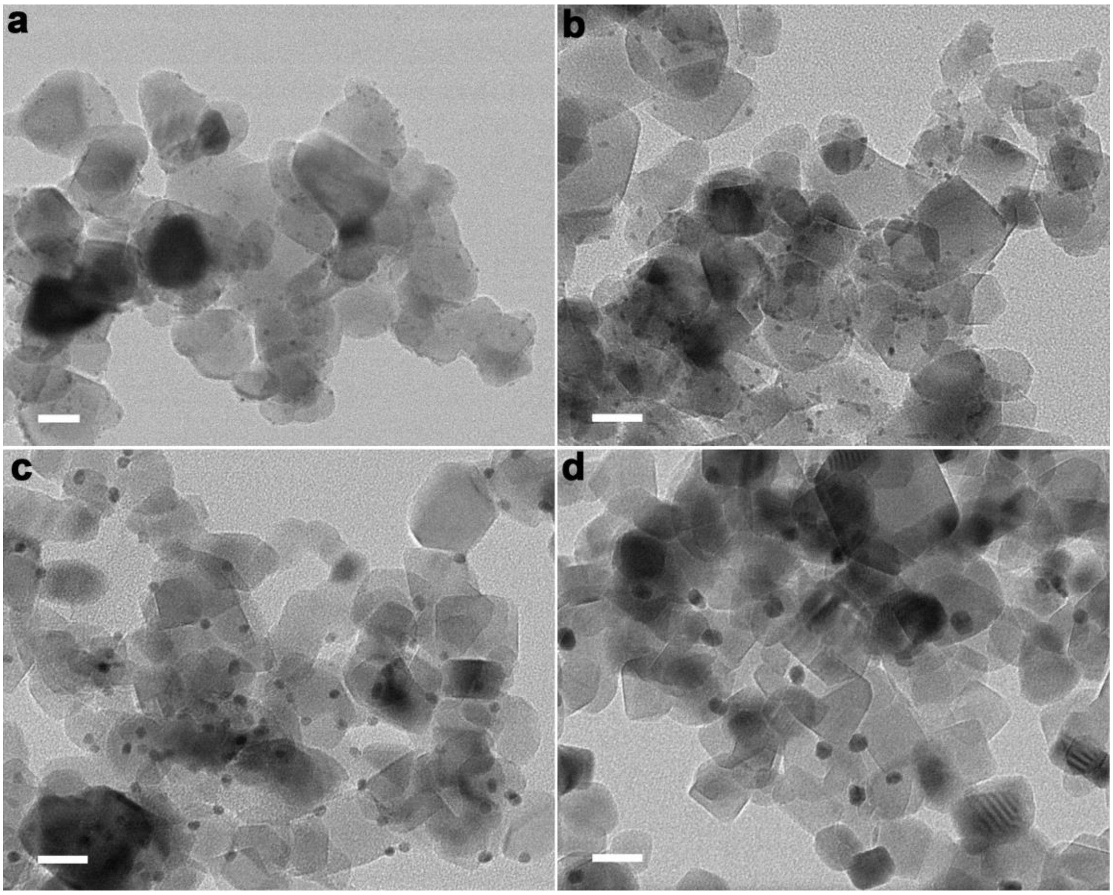

Supplementary Figure 3 | TEM analysis. HR-TEM images of **a** 1.8nmPt/TiO_2; **b** 3.4nmPt/TiO_2; **c** 4.3nmPt/TiO_2 and **d** 7.0nmPt/TiO_2. The scale bars are 20nm in **a-d**.

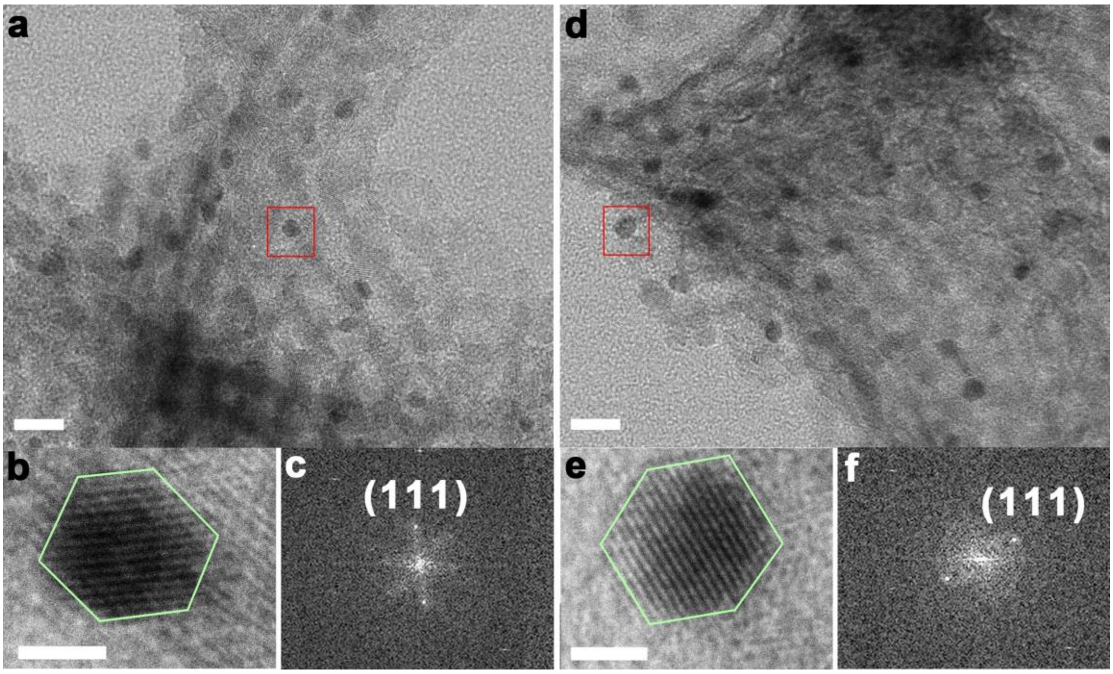

Supplementary Figure 4 | Structural characterizations of Pt NPs. a, d HR-TEM images of 3.4PHTSO and 4.3PHTSO, where the red squares represent the local magnification of one Pt NP **b, e**, the marked facets show the classic truncated octahedron shape of Pt NP. **c, f** The corresponding FFT patterns of Pt NP. The scale bars are 10nm in **a, d** and 2nm in **b, e**.

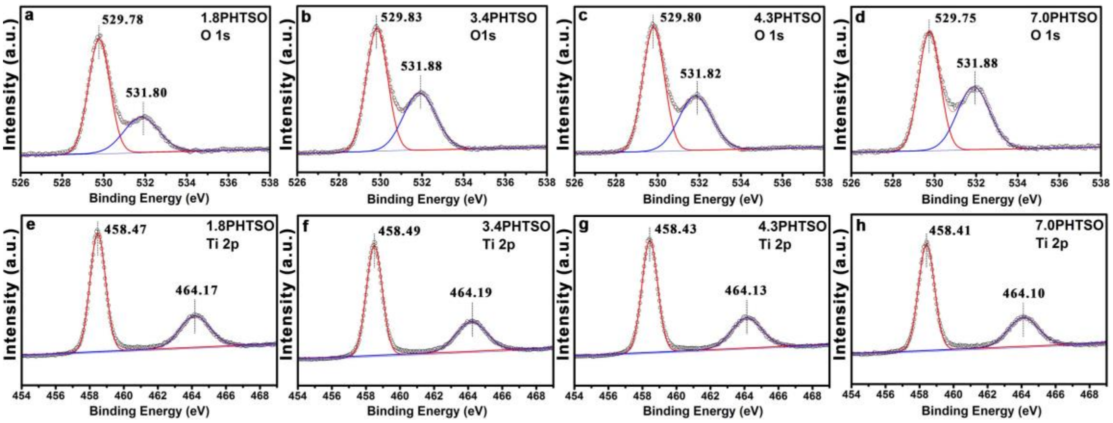

Supplementary Figure 5 | XPS analysis. High-resolution O 1s and Ti 2p XPS spectra of **a, e** 1.8PHTSO, **b, f** 3.4PHTSO, **c, g** 4.3PHTSO and **d, h** 7.0PHTSO.

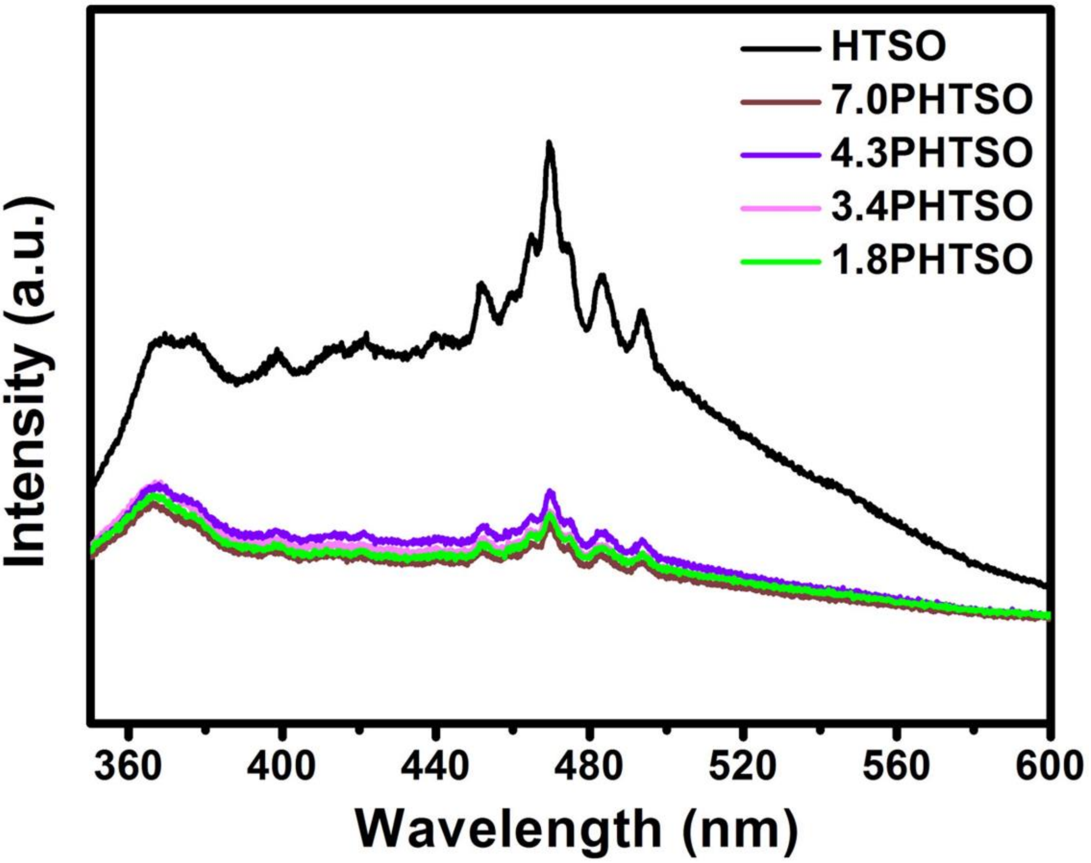

Supplementary Figure 6 | Room temperature photoluminescence emission spectra of *x*PHTSO (*x* = 1.8, 3.4, 4.3 and 7.0), where the excitation wavelength is fixed at 315 nm.

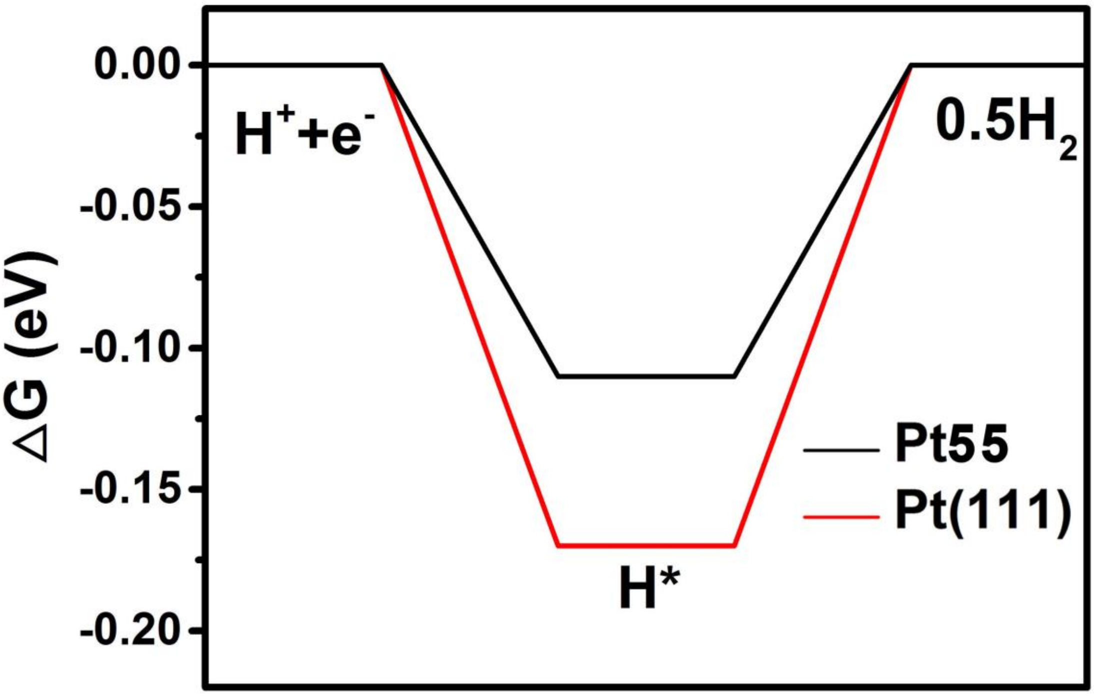

Supplementary Figure 7 | Calculated free energy diagram for H^+ reduction to H_2 by the thermochemical model on Pt(111) surface and Pt55.

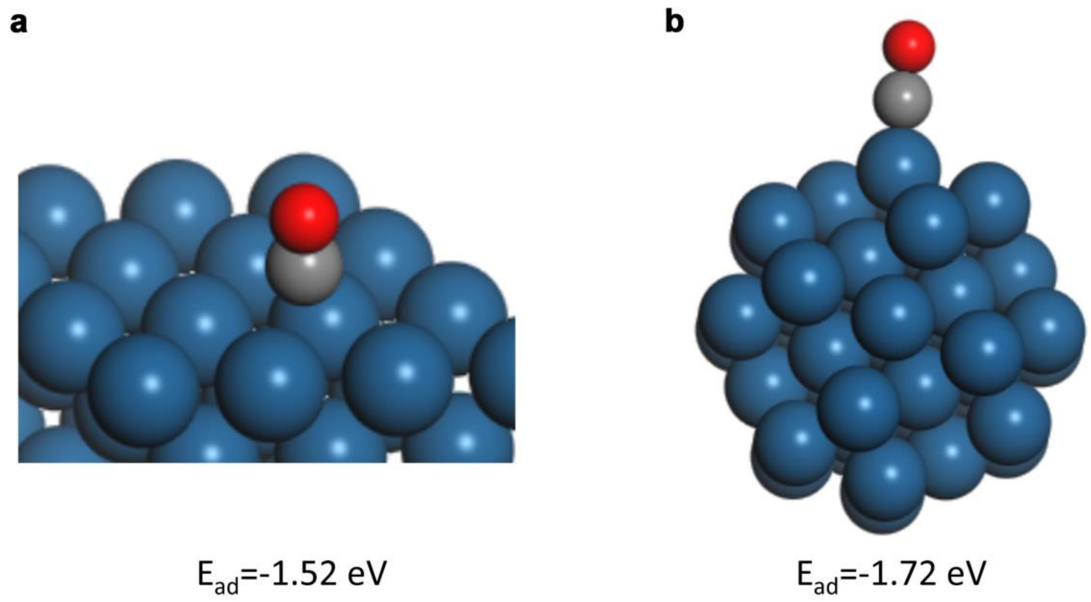

Supplementary Figure 8 | Adsorption energy of CO on **a** Pt(111), **b** Pt55.

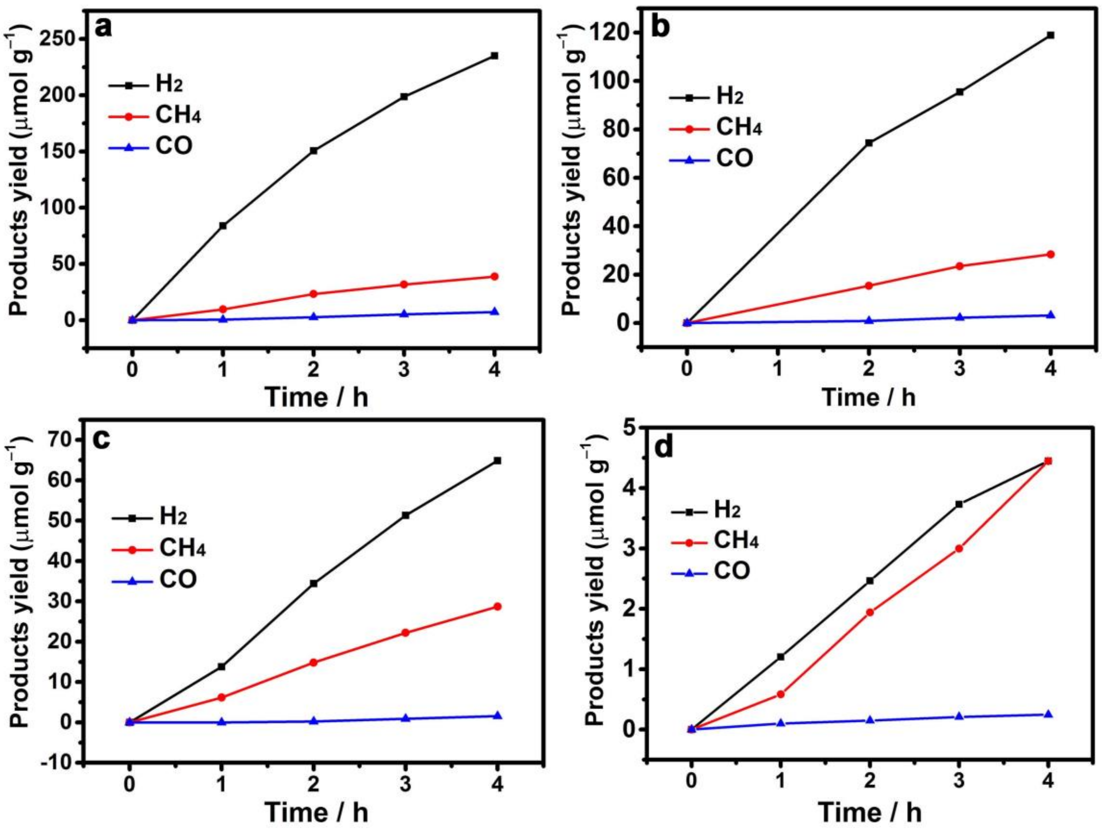

Supplementary Figure 9 | Products yield evaluations of the *x*PHTSO. Time course evolution of CH_4, CO and H_2 production in 4 hours over different samples: **a** 1.8PHTSO, **b** 3.4PHTSO, **c** 4.3PHTSO and **d** 7.0PHTSO.

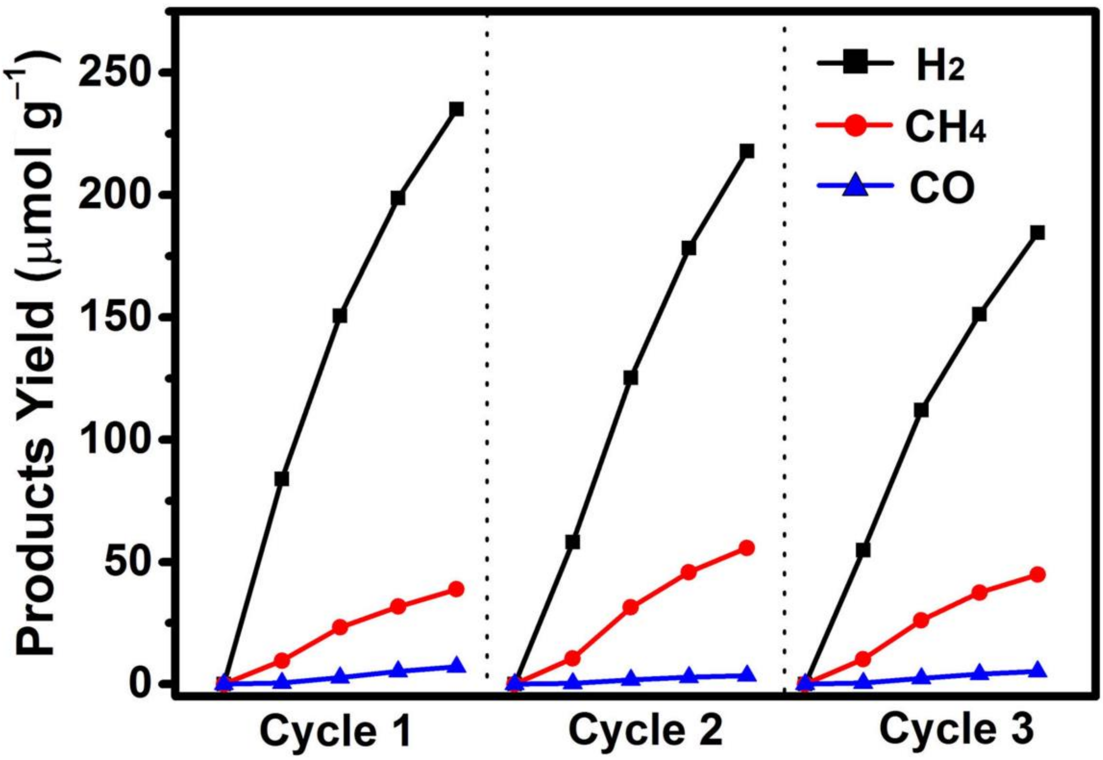

Supplementary Figure 10 | Cyclic experiments of CO2PR over 1.8PHTSO (irradiation for 4 hours each cycle)

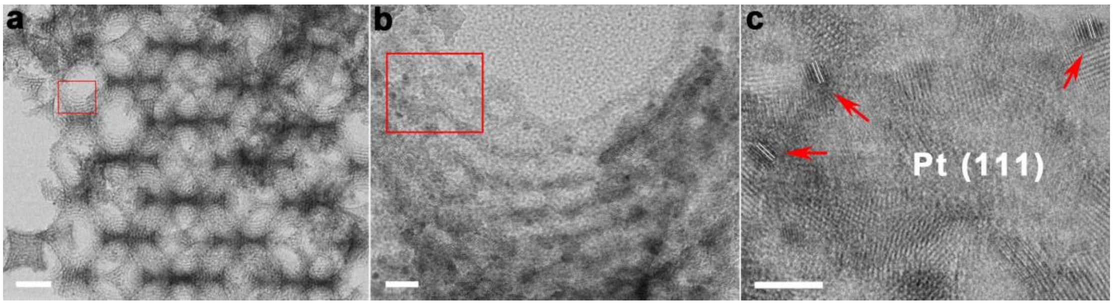

Supplementary Figure 11 | TEM analysis. TEM and corresponding HR-TEM images of 1.8PHTSO after CO pulse adsorption and 280 °C He flow sweep, the red squares indicate the stepwise magnification of local sites, the red arrows point out the dispersive Pt NPs. The scale bar is 100nm in **a**, 10nm in **b** and 5nm in **c**.

Supplementary Table 1 | N_2 physisorption characterization of *x*PHTSO (*x* = 1.8, 3.4, 4.3 and 7.0)

**Samples**	**Mean pore size (nm)**	**S_{BET} (m^2 g^{-1})**
1.8PHTSO	4.8	138.9
3.4PHTSO	5.5	224.8
4.3PHTSO	5.6	229.2
7.0PHTSO	5.6	208.3

Supplementary Table 2 | Calculation formulas of different surfaces sites' numbers based on one truncated octahedron model

Total atoms' number of one particle (N_T)	$16m^3-33m^2+24m-6$
Total surface atoms of one particle (N_S)	$30m^2-60m+32$
Corner atoms of one particle (N_C)	24
Edge atoms of one particle (N_E)	$36(m-2)$
Total (100) facets atoms of one particle	$6(m-2)^2$
Total (111) facets atoms of one particle	$8(3m^2-9m+7)$

See Supplementary Note 2 for more information.

Supplementary Table 3 | Peak information summary from high-resolution Pt 4f XPS spectra of *x*PHTSO (*x* = 1.8, 3.4, 4.3 and 7.0).

Peak Info / Sample	FWHM	Peak Area	Peak Center (eV)	Peak Type	Area Fraction (%)
1.8PHTSO	1.08	810	70.7 (Pt 4f 7/2 Pt^0)	Gaussian	Pt^0 (69.8) Pt^{2+} (16.8) Pt^{4+} (13.3)
	0.94	150	71.6 (Pt 4f 7/2 Pt^{2+})		
	1.40	100	72.4 (Pt 4f 7/2 Pt^{4+})		
	1.08	660	74.0 (Pt 4f 5/2 Pt^0)		
	0.95	205	75.0 (Pt 4f 5/2 Pt^{2+})		
	1.4	180	76.0 (Pt 4f 5/2 Pt^{4+})		
3.4PHTSO	0.98	1004	70.4 (Pt 4f 7/2 Pt^0)	Gaussian	Pt^0 (67.3) Pt^{2+} (14.0) Pt^{4+} (18.7)
	0.90	260	71.3 (Pt 4f 7/2 Pt^{2+})		
	1.5	143	72.4 (Pt 4f 7/2 Pt^{4+})		
	1.03	940	73.7 (Pt 4f 5/2 Pt^0)		
	0.90	300	74.7 (Pt 4f 5/2 Pt^{2+})		
	1.5	241	75.8 (Pt 4f 5/2 Pt^{4+})		
4.3PHTSO	1.01	760	70.4 (Pt 4f 7/2 Pt^0)	Gaussian	Pt^0 (67.1) Pt^{2+} (19.3) Pt^{4+} (13.6)
	0.90	220	71.3 (Pt 4f 7/2 Pt^{2+})		
	1.40	103	72.3 (Pt 4f 7/2 Pt^{4+})		
	1.05	726	73.7 (Pt 4f 5/2 Pt^0)		
	0.90	227	74.7 (Pt 4f 5/2 Pt^{2+})		
	1.40	180	75.8 (Pt 4f 5/2 Pt^{4+})		
7.0PHTSO	0.99	270	70.3 (Pt 4f 7/2 Pt^0)	Gaussian	Pt^0 (67.1) Pt^{2+} (18.7) Pt^{4+} (14.2)
	0.96	60	71.1 (Pt 4f 7/2 Pt^{2+})		
	1.5	35	71.8 (Pt 4f 7/2 Pt^{4+})		
	0.98	250	73.6 (Pt 4f 5/2 Pt^0)		
	0.96	85	74.6 (Pt 4f 5/2 Pt^{2+})		
	1.5	75	75.6 (Pt 4f 5/2 Pt^{4+})		

Supplementary Table 4 | Thermodynamic data of gas-phase species. Zero-point energies (ZPE) are calculated with experimental vibrational data, the integrated heat capacity (δH_0) and entropy (S) at 298.15K are obtained from reference. For water, the entropy is calculated at 0.035bar, because at this pressure gas-phase H_2O is in equilibrium with liquid water at 298.15K.

**Adsorbate**	**E_{elec} (ev)**	**ZPE(ev)**	**δH_0(ev)**	**-TS(ev)**	**μ(ev)**
H_2	-0.00	0.27	0.09	-0.41	-0.05
CO	1.75	0.13	0.09	-0.62	1.35
CO_2	0.9	0.31	0.10	-0.66	0.65
H_2O	0.03	0.56	0.10	-0.68	0.01
CH_4	-1.22	1.20	0.10	-0.65	-0.56

Supplementary Table 5 | Contributions to the adsorbate free energy on Pt(111) from the zero-point energy correction, enthalpic temperature correction, entropy and the total free energy correction respectively. ΔE is the electronic energy of the state minus the electronic energy of the clean slab(s) associated with that state.

**Adsorbate**		**ΔE (ev)**	**ZPE(ev)**	**δH_0(ev)**	**-TS(ev)**	**ΔG(ev)**
*COOH	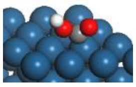	0.43	0.60	0.01	-0.18	0.24
*CO	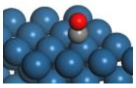	0.23	0.18	0.01	-0.15	-0.55
*COH	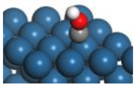	1.17	0.44	0.01	-0.18	-0.15
*CHOH	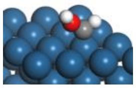	1.16	0.76	0.01	-0.19	0.19
*CH	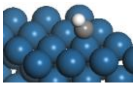	1.10	0.47	0.01	-0.17	-0.41
*CH_2	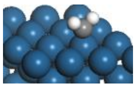	0.67	0.49	0.01	-0.17	-0.34
*CH_3	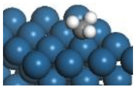	-0.08	0.50	0.01	-0.17	-0.68

Supplementary Table 6 | Contributions to the adsorbate free energy on Pt55 from the zero-point energy correction, enthalpic temperature correction, entropy and the total free energy correction respectively. ΔE is the electronic energy of the state minus the electronic energy of the clean slab(s) associated with that state.

**Adsorbate**		**ΔE (ev)**	**ZPE(ev)**	**δH_0(ev)**	**-TS(ev)**	**ΔG(ev)**
*COOH	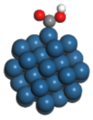	0.35	0.60	0.01	-0.18	0.16
*CO	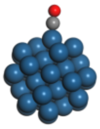	0.03	0.18	0.01	-0.15	-0.64
*COH	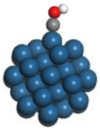	1.24	0.44	0.01	-0.18	-0.01
*CHOH	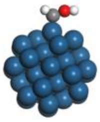	1.28	0.76	0.01	-0.19	0.32
*CH	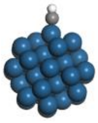	1.02	0.47	0.01	-0.17	-0.46
*CH_2	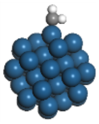	0.61	0.49	0.01	-0.17	-0.37
*CH_3	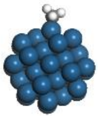	-0.16	0.50	0.01	-0.17	-0.72

Supplementary Note 1

Firstly, we would like to explain the saying of “close contact between the Pt and TiO_2”. In fact, different from the PVP-stabilized Pt NPs supported on HTSO, the ligand-free Pt NPs supported on HTSO obtained through our synthesis strategy would result in the generation of direct metal-oxide contact interface (Figs. 1j~m), which is beneficial to the ready photo-generated electrons’ separation and transfer. What’s more, there’s no doubt that the metal-oxide interface plays important roles in photocatalysis, prior studies have shown that when metal NPs and semiconductor contacted electrically (usually the work function of metal is higher than that of semiconductor), the conduction band of semiconductor bending upward and accompanied with the migration of electrons to the metal until their Fermi levels are aligned. In this case, the Schottky barrier could be formed at the metal-semiconductor interface to promote the photo-generated electrons’ accumulation at the metal sites and prevent the recombination of electron-hole pairs[1, 2]. So, in our case, the Pt and HTSO (TiO_2) represent the metal and semiconductor respectively and we have also added more discussion about the electronic properties of the interface between Pt-TiO_2 in the revised manuscript.

According to previous studies, generally, the strong interactions could be generated between the group VIII metals and the reducible metal-oxide supports (such as TiO_2) in H_2 atmosphere at a high temperature[3, 4]. Subsequently, the formed strong metal-support interactions (SMSI) would result in two the distinct features: 1) electrons transferred from the support to the metal sites and result in an increased

electron density at the metal sites; 2) the adsorption towards CO and H_2 at the metal sites decreased dramatically due to the encapsulation of metal NPs by the support[5, 6]. Amiridis et al. once studied the effects of reduction temperature and SMSI on Pt/TiO_2 composites, the results indicate that the H_2 pre-treatment of Pt/TiO_2 at a low reduction temperature (200 ^oC or less) cannot affect its CO adsorption behavior, however, when H_2 pre-treatment of Pt/TiO_2 exceeds 300 ^oC, the adsorption amount towards CO could be reduced dramatically.[7] Therefore, in our case, consider of the relative consistent and gentle preparation method of the *x*PHTSO (*x* = 1.8, 3.4, 4.3 and 7.0), the reduction temperature of Pt precursor is only 160 ^oC in ethylene glycol (EG), hence, it can be eliminated the SMSI effect between Pt and HTSO. Besides, CO-pulse adsorption result indicates the adsorption amount of CO is highly dependent on the size value of Pt NPs, in addition, the calculated size values based on the adsorption result are correlated with the result from the TEM measurement. These results could demonstrate the Pt NPs are not encapsulated by the support and the mutual interactions are not strong like the SMSI effect. To further exclude this possibility in our case, the high-resolution O 1s and Ti 2p XPS spectra of *x*PHTSO (*x* = 1.8, 3.4, 4.3 and 7.0) were added in Supplementary Fig. 5. The result shows that the binding energies of lattice O and Ti possess almost the same value with the size variation of Pt NPs, which indicates the interactions between Pt NPs and TiO_2 in *x*PHTSO (*x* = 1.8, 3.4, 4.3 and 7.0) are equivalent, increase or decrease the size of Pt NPs couldn't change the mutual interactions between Pt and TiO_2[8, 9]. On the contrary, with the decrease of the size of Pt NPs, the obvious red-shift of Pt 4f binding energy to a

higher region further indicates the binding energy alterations of Pt 4f in the *x*PHTSO result from the geometric properties of Pt NPs instead of the different metal-support interactions (Figs. 3a-d). Hence, we can eliminate the SMSI effect from our case, the size effect of Pt NPs is the main reason for the activity and selectivity variation in CO2PR.

Supplementary Note 2

The m in the formula denotes as the number of atoms lying on an equivalent edge (corner atoms included), which is calculated based on the equation of d (nm, size of Pt NPs) = 1.105 * $N_T^{1/3}$ * $d_{Pt\ atom}$, the size of one Pt atom ($d_{Pt\ atom}$) is 0.276nm.[10, 11] Therefore, the m value for the size at 1.8nm, 3.4nm, 4.3nm and 7.0nm Pt NPs is 3.0176, 5.1053, 6.2778 and 9.7931 respectively.

Supplementary References

1. Amy L. Linsebigler, Guangquan Lu, John T. Yates J. Photocatalysis on TiO_2 surfaces: principles, mechanisms, and selected results. *Chem. Rev.* **95**, 735-758 (1995).
2. Habisreutinger SN, Schmidt-Mende L, Stolarczyk JK. Photocatalytic reduction of CO_2 on TiO_2 and other semiconductors. *Angew. Chem. Int. Ed.* **52**, 7372-7408 (2013).
3. S. J. Tauster, S. C. Fung, Garten RL. Strong Metal-Support Interactions. Group 8 Noble Metals Supported on TiO_2. *J. Am. Chem. Soc.* **100**, 170 (1978).
4. S. J. Tauster, S. C. Fung, R. T. K. Baker, Horsley JA. Strong interactions in supported-metal catalysts. *Science* **211**, 1121 (1981).
5. Matsubu JC, *et al.* Adsorbate-mediated strong metal-support interactions in oxide-supported Rh catalysts. *Nat. Chem.* **9**, 120-127 (2017).
6. Liu X, *et al.* Strong metal-support interactions between gold nanoparticles and ZnO nanorods in CO oxidation. *J. Am. Chem. Soc.* **134**, 1121-1125 (2012).
7. Oleg S. Alexeev, Soo Yin Chin, Mark H. Engelhard, Lorna Ortiz-Soto, Amiridis MD. Effects of reduction temperature and metal-support interactions on the catalytic activity of Pt/γ-Al_2O_3 and Pt/TiO_2 for the oxidation of CO in the presence and absence of H_2. *J Phys Chem B* **109**, 23430-23443 (2005).
8. Wangcheng Zhan, *et al.* A sacrificial coating strategy toward enhancement of metal−support interaction for ultrastable Au nanocatalysts. *J. Am. Chem. Soc.* **138**, 16130-16139 (2016).
9. Xing Cheng, *et al.* Highly active, stable oxidized platinum clusters as electrocatalysts for the hydrogen evolution reaction. *Energy Environ. Sci.* **10**, 2450-2458 (2017).
10. Chen W, *et al.* Mechanistic insight into size-dependent activity and durability in Pt/CNT catalyzed hydrolytic dehydrogenation of ammonia borane. *J. Am. Chem. Soc.* **136**, 16736-16739 (2014).
11. Hardeveld RV, Hartog F. The statistic of surface atoms and surface sites on metal crystals. *Surf. Sci.* **15**, 189-230 (1969).
